# Large Language Model Risk of Bias Assessment for Studies in Environmental Health: Proof-of-Concept Study

**DOI:** 10.2196/99716

**Published:** 2026-11-09

**Authors:** Gaelen P Adam, Eitan Agai, Riaz Qureshi, Naeem Meman, Stephanie Qureshi, Winifred W Yu, Juleen Lam, Karen A Robinson

**Affiliations:** 1 Center for Evidence Synthesis in Health, Department of Health Services, Policy, and Practice, Brown University School of Public Health, 121 South Main St, Providence, RI, 02903, United States, 1 617-470-5685; 2 PICO Portal, St. Petersburg, FL, United States; 3 Department of Ophthalmology, School of Medicine, University of Colorado Anschutz Medical Campus, Aurora, CO, United States; 4 Department of Epidemiology, School of Public Health, University of Colorado Anschutz Medical Campus, Aurora, CO, United States; 5 Department of Public Health, California State University, East Bay, Hayward, CA, United States; 6 Department of Medicine, School of Medicine, Johns Hopkins University, Baltimore, MD, United States

**Keywords:** systematic review methods, large language models, risk of bias assessment, environmental health, AI

## Abstract

**Background:**

The assessment of the risk of bias (RoB) of each study is a necessary but labor-intensive step in conducting a systematic review (SR). There is a potential for AI to reduce the time and effort needed for RoB assessment and improve consistency between reviewers, but little is known about the accuracy of AI-based RoB assessments.

**Objective:**

In this study, we aim to evaluate the performance of a large language model (LLM) when given the questions from the RoB tool and the relevant passages from the full-text papers in assessing RoB in observational studies of environmental exposures and health outcomes.

**Methods:**

We evaluated the performance of GPT-5-mini on 128 observational studies from a SR of per- and polyfluoroalkyl substances and health outcomes conducted by the National Academies of Sciences. The navigation guide (NavGuide), a domain-based RoB tool designed for environmental studies, was used. For each paper-domain pair, the LLM was provided with the questions and guidance from the NavGuide and the human-identified text passages addressing that domain. The LLM returned a structured RoB rating, which was compared to the human-adjudicated ratings to quantify agreement and identify patterns of discrepancy.

**Results:**

GPT-5-mini RoB assessments for exact matches (64.5%; κ=0.199, Gwet AC2=0.882, *F*_1_-score=0.311) remained lower than the concordance between humans and the reference standard (87.5%-91.4%). Performance improved substantially for partial matches, reflecting agreement in direction but not necessarily in magnitude (eg, “low” and “probably low”), with percent concordance of 91.9% (κ=0.241, Gwet AC2=0.910, *F*_1_-score=0.614; for humans, 97.9%-98.6% concordance). Performance varied across the NavGuide domains, with the worst performance in domain 1 (selection bias) and the best in domain 4 (outcome assessment). The models tended to be more stringent than humans, often assigning higher RoB ratings (eg, “probably low” when humans assigned “low”).

**Conclusions:**

Our results suggest that although concordance was moderate and inconsistent across domains, LLMs may be sufficiently accurate for use with human oversight. LLMs can flag cases for human adjudication or consistency checking but are not ready for use as an independent second reviewer without human oversight.

## Introduction

### Background

Systematic reviews (SRs) and similar types of evidence synthesis are considered the gold standard for assessing evidence used in decision-making across health contexts. In environmental health research, SRs are conducted to critically assess and consolidate large amounts of observational data on environmental exposures and their impact on human health.

One of the necessary steps in conducting an SR is the critical appraisal, or assessment, of the methodological quality of the included studies. Reviewers assess the likelihood that any given study could be biased, a concept referred to as the risk of bias (RoB). The existence of such bias can potentially skew the results of the SR. Observational studies are increasingly being included in SRs and are the norm in SRs of environmental exposures [1,2]. However, observational studies have specific methodological challenges [3,4].

Several tools have been developed to assess RoB in observational studies, including the Newcastle-Ottawa Scale [5], Risk of Bias in Nonrandomized Studies of Interventions (ROBINS-I) [2], and Risk of Bias in Nonrandomized Studies of Exposure (ROBINS-E) [6]. The navigation guide (NavGuide) methodology was developed specifically for evidence synthesis in environmental exposures and health-related outcomes, designed to provide a rigorous and transparent approach for evaluating RoB of studies on environmental exposures and health outcomes [1].

Manual reviewer assessment of RoB is often time- and resource-intensive, as well as prone to inconsistencies [7]. Incorporating AI, specifically when integrated into human workflows, may reduce time and effort and improve consistency [8]. Several studies have evaluated RoB assessment by large language models (LLMs) [9-16], but few have evaluated these models in studies other than randomized controlled trials (RCTs), and in those that have, results have been inconsistent [17,18]. Thus, while RoB is an area of potential for the integration of LLMs into evidence synthesis processes, more research is needed to evaluate their performance, particularly in studies that incorporate this new technology in human workflows and in studies other than RCTs.

### Purpose of the Study

In this study, we aim to focus on the RoB assessment step: how well can LLMs reproduce expert RoB judgments when given the same text passages from the full-text papers that human reviewers used in assessing RoB in observational studies of environmental exposures and health outcomes across 8 of the 9 domains of the NavGuide?

## Methods

### Study Design

We evaluated the performance of 2 LLMs (GPT-5-mini and Google Gemini-3) for RoB assessment. For each paper-domain pair, the LLM was provided with human-identified text passages addressing that domain and returned a structured RoB rating using the 5-level scale set out in the NavGuide [1]. These ratings were then compared with the human-adjudicated ratings to quantify concordance and identify patterns of discrepancy. The protocol was prospectively registered through the Open Science Framework [19].

### Ethical Considerations

This research did not include human participants.

### Reference Standard Creation

The reference standard for this study consisted of 128 full-text papers from an SR on per- and polyfluoroalkyl substances (PFAS) and health outcomes, which was used to inform the National Academies of Sciences (NAS) 2022 Guidance on PFAS exposure, testing, and clinical follow-up [20]. These papers comprise the open-access full-text papers of the 139 studies that were included in the published NAS review. We included only open-access publications to ensure access to the full texts and to avoid infringing on copyright laws. The papers were identified through a comprehensive database search of MEDLINE, Embase (both via the Ovid interface), and Scopus in March 2021. Full details of the NAS review methods are available in the report [20].

For this project, we developed a structured form based on the NavGuide and retrieved the full-text papers of the 128 studies. The form covers 8 of the 9 human RoB domains (all except the “other” category) in the NavGuide, with the signaling questions and response options [1]. We used the detailed set of instructions for assessing RoB in this topic, including explanations of the details required for each level of RoB rating for each domain, developed by the authors of the NAS report. The full guide is included in Multimedia Appendix 1. The 5 options for rating levels are “high,” “probably high,” “probably low,” “low,” and “not applicable.” Table 1 provides a brief description of each domain, and the full guide is available in Multimedia Appendix 1. Two experienced reviewers independently assessed RoB in all 128 papers across each of the NavGuide domains, providing both the rationales for their assessments and extracted passages from the full-text papers that they used in making their assessments. The passages extracted by the reviewers were combined to form the relevant text passages provided to the LLM; the LLMs were not provided with access to the reviewers’ rationale or the full-text papers. The LLMs were only asked to assess the first 8 domains, as domain 9 addresses “Other potential sources of bias” and would be impossible to standardize.

**Table 1.** NavGuide risk of bias domains.

Domain	Name	Question
1	Selection bias	Are the study groups at risk of not representing their source populations in a manner that might introduce selection bias?
2	Blinding	Was knowledge of the group assignments inadequately prevented (ie, blinded or masked) during the study, potentially leading to subjective measurement of either exposure or outcome?
3	Exposure assessment	Were exposure assessment methods lacking accuracy?
4	Outcome assessment	Were outcome assessment methods lacking accuracy?
5	Confounding	Was potential confounding inadequately incorporated?
6	Incomplete outcome data	Were incomplete outcome data inadequately addressed?
7	Selective outcome reporting	Does the study report appear to have selective outcome reporting?
8	Conflict of interest	Did the study receive any support from a company, study author, or other entity having a financial interest in any of the exposures studied?
9	Other issues	Did the study appear to have other problems that could put it at a risk of bias?

### Models and Tasks

We presented each LLM with the same guidance that was given to the human reviewer, along with a generic prompt. The guidance was also identical to that used by the authors of the NAS report. It included the RoB questions, as well as specific criteria on how to assess RoB for that question and guidelines to determine the RoB rating level, as they appear in the NavGuide. Specific modifications were made by a subject-matter expert on PFAS (eg, serum PFAS levels as an example of biomonitoring data). In addition to the full guidance, we also presented each LLM with the relevant passages identified by the human reviewers for each domain’s assessment.

All LLM assessments were conducted with GPT-5-mini using Azure OpenAI (version 2025-08-07) [21] and (as a sensitivity analysis) Google Gemini-3 using OpenAI’s compatibility layer for Gemini (January 2026) [22], independently, in a zero-shot configuration (ie, no additional training was done before providing the passages for assessment). Hereafter, we will refer to the models as GPT-5-mini and Gemini-3. Default model settings for reasoning effort and verbosity were used consistently across all assessments. The implementation used structured output, and the schema for the output was in the Python code. The API was used rather than the web interface. The LLM was asked to return a RoB rating for each paper-domain pair and provide its justification (ie, a structured query where the LLM is asked to provide both an answer and a justification of why it chose that answer [23,24]). Full details and prompts are provided in Multimedia Appendix 1.

### Evaluation

The primary evaluation focused on the initial set of ratings from GPT-5-mini. To evaluate performance, human reviewers compared the responses from GPT-5-mini to the human consensus judgment and reviewed the LLM’s justification when there was disagreement. The 3 options for this assessment were as follows:

- Exact match: LLM and human consensus labels are identical
- Acceptable agreement: LLM and human ratings agree in direction but not in magnitude (eg, “low” vs “probably low” or “high” vs “probably high”)
- Disagreement: any other mismatch (eg, “low” vs “probably high” or “high”)

In cases other than an exact match, human reviewers (GA, SQ, and WY) evaluated the justifications from the original human reviewer and those from the LLM, documenting any issues, gaps, or agreements. We calculated percent concordance for both exact matches and partial matches (exact match or agreement in direction). The purpose of the partial-match analysis was to assess whether AI performance fell within the range of disagreement typically observed between expert human reviewers, rather than to imply that these are interchangeable in all contexts. Disagreements between low and probably low, or high and probably high, would be unlikely to materially alter overall review conclusions in most cases, whereas larger discrepancies could have a more meaningful impact and be of greater concern.

We evaluated performance for each of the 8 domains and overall, as well as in subgroups of interest (eg, human-rated “high” vs “low” RoB). We also calculated the following concordance measures for overall performance: weighted Cohen κ [25], using quadratic weights to correct for chance agreement in cases where the differences between categories differ in importance [26]; Gwet AC2 [27], which also corrects for chance concordance but is more stable with imbalanced data; and a macro *F*_1_-score, which is unweighted by category size, to compare raters. We conducted all analyses in R (version 4.3.3; R Foundation for Statistical Computing) using the irrCAC package; Claude Sonnet 5.5 Medium (July 28, 2026) assisted with code writing, but all code was verified by a human team member (GA). For interpretation, we used the following scale for the κ [28] and Gwet AC2 [29] scores: <0.20: poor; 0.21‐0.40: fair; 0.41‐0.60: moderate; 0.61‐0.80: substantial; 0.81‐1.00: almost perfect. The performance of a second LLM (Gemini-3) was evaluated as a sensitivity analysis to assess whether the results would generalize across models. Finally, we assessed how well the LLMs could retrieve relevant passages from full-text PDFs and how well they could assess RoB based on the passages they identified. These assessments were automated, and we did not perform any evaluation of the justifications.

For the primary (GPT-5-mini) analysis, we used a descriptive evaluation of the human and LLM justifications to determine if there were any patterns in the differing assessments that could point to ways to improve the quality of LLM-assisted RoB assessment. Three team members (GA, SQ, and WY) reviewed each citation where the LLM and human consensus did not exactly match and narratively described the discrepancy based on the LLM and human justifications. A subject expert (JL) then evaluated these descriptions to identify themes or patterns that might explain the discrepancies and to reassess the consensus RoB. A second reviewer (GA) assessed the documentation of the acceptable agreement labels, categorizing them into a set of tags. From this analysis, 4 team members (JL, SQ, WY, and GA) derived a set of themes to describe possible sources of discrepancies in assessment between humans and GPT-5-mini. However, it is important to note that the LLM-generated justifications are not validated indicators of the model’s internal reasoning process and should be interpreted as generated text outputs rather than reliable explanations of how the model arrived at its conclusions.

## Results

### Citations Assessed and Estimated Costs

The LLMs were able to assess RoB in most (124-128) of the citations across the 8 RoB domains as defined by the NavGuide. Twelve citations could not be assessed for at least one domain due to the lack of human-specified passages or incorrectly formatted passages (ie, quotation marks were missing). Results are presented only for the citations in each domain that were assessed by the LLM (a total of 1007 paper-domain pairs). The human RoB consensus rating was “low” for 924 paper-domain pairs, “probably low” for 58 pairs, and “probably high” for 25 pairs. No paper-domain pair was assessed as “high” RoB. Each assessment used approximately 15,000 input tokens, including the full-text PDF, system instructions, domain questions and their descriptions, and the associated prompt, plus approximately 1000 output tokens, including reasoning. Based on these assumptions, the estimated LLM cost was approximately US $0.25 per paper using GPT-5-mini and US $0.011 per paper using Gemini-3 Flash.

### Overall Performance

For exact matches, GPT-5-mini achieved 64.5% (647/1003) concordance with the human consensus. Concordance improved substantially for partial matches (ie, agreement in direction but not necessarily in magnitude, such as “low” and “probably low”), with GPT-5-mini achieving 91.4% (922/1006) concordance with the human consensus. The quadratic-weighted Cohen κ values for exact matches were low (0.199), indicating poor concordance beyond chance. For partial matches, κ values improved but remained fair at best (0.241). However, this may be a result of the imbalanced data, as the κ values were also very low for the concordance between human raters. The Gwet AC2, which is less sensitive to imbalanced data, indicated nearly perfect performance for exact (0.882) and partial matches (0.910). Finally, the macro-averaged *F*_1_-scores, which count rare and common categories equally, were 0.311 for exact matches and 0.614 for partial matches. The results for Gemini-3 were similar, with somewhat poorer performance for exact matches and better performance for partial matches. Details are given in Tables 2 and 3, and full confusion matrices for each model are included in Multimedia Appendix 1. The concordance between human reviewers and between each human and the consensus was higher than AI-human concordance. The 2 human reviewers, while reviewing 1007 paper-domain pairs, showed 81.2% (n=817) exact concordance with each other and 97.5% (n=981) partial match. Individual human reviewers achieved 87.5% (n=881) to 91.4% (n=919) exact match with the final human consensus and 97.9% (n=986) to 98.6% (n=992) partial match. Weighted Cohen κ ranged from 0.399 to 0.597, Gwet AC2 from 0.975 to 0.984, and *F*_1_-scores from 0.449 to 0.821. The rest of the results are given as percent concordance.

**Table 2.** Overall results for each large language model and humans: exact match.^a^

Comparison	Number of citations, n	Percent concordance (95% CI)	Gwet AC2 (95% CI)	Cohen κ (95% CI)	*F*_1_-score (95% CI)
GPT-5-mini	1003	64.5 (61.5-67.5)	0.882 (0.863-0.902)	0.199 (0.136-0.262)	0.311 (0.269-0.347)
Gemini-3	1007	50.7 (47.7-53.8)	0.891 (0.880-0.903)	0.164 (0.102-0.226)	0.250 (0.212-0.297)
Human 1	1007	87.5 (85.4-89.5)	0.975 (0.969-0.981)	0.399 (0.286-0.513)	0.449 (0.394-0.650)
Human 2	1006	91.4 (89.6-93.1)	0.984 (0.980-0.989)	0.597 (0.497-0.696)	0.527 (0.475-0.754)
Human 1-Human 2	1006	81.2 (78.8-83.6)	0.964 (0.957-0.971)	0.138 (0.044-0.232)	0.322 (0.269-0.457)

a N<1007 in cases where assessments of not applicable were removed.

**Table 3.** Overall results for each large language model (LLM) and humans: partial match.^a^

Comparison	Number of citations, n	Percent concordance (95% CI)	Gwet AC2 (95% CI)	Cohen κ (95% CI)	*F*_1_-score (95% CI)
GPT-5-mini	1003	91.9 (90.1-93.5)	0.910 (0.889-0.930)	0.241 (0.133-0.349)	0.614 (0.557-0.669)
Gemini-3	1007	95.6 (94.2-96.8)	0.954 (0.939-0.968)	0.269 (0.118-0.419)	0.634 (0.553-0.704)
Human 1	1007	97.9 (96.8-98.7)	0.978 (0.969-0.988)	0.478 (0.287-0.668)	0.739 (0.632-0.830)
Human 2	1006	98.6 (97.7-99.2)	0.986 (0.978-0.993)	0.643 (0.469-0.817)	0.821 (0.724-0.898)
Human 1-Human 2	1006	97.5 (96.6-98.5)	0.974 (0.964-0.985)	0.181 (−0.008-0.370)	0.590 (0.494-0.683)

a Partial match means an agreement in direction but not necessarily in magnitude (eg, low+probably low); n<1007 in cases where assessments of not applicable were removed.

In general, the LLM was more stringent than human reviewers, frequently assigning “probably low” RoB when humans assigned “low” risk, or “high” risk when humans assigned “probably high” risk. In only 2.9% (29/1003) of assessments, GPT-5-mini assigned a less stringent rating; it was more stringent 32.6% (327/1003) of the time and the same 64.5% (647/1003) of the time. Gemini-3 showed similar results. This tendency to be more stringent may have contributed to the discrepancy between the LLM’s exact match and partial match performance. Full results are provided in Figure S1 of Multimedia Appendix 1.

**Figure 1.**
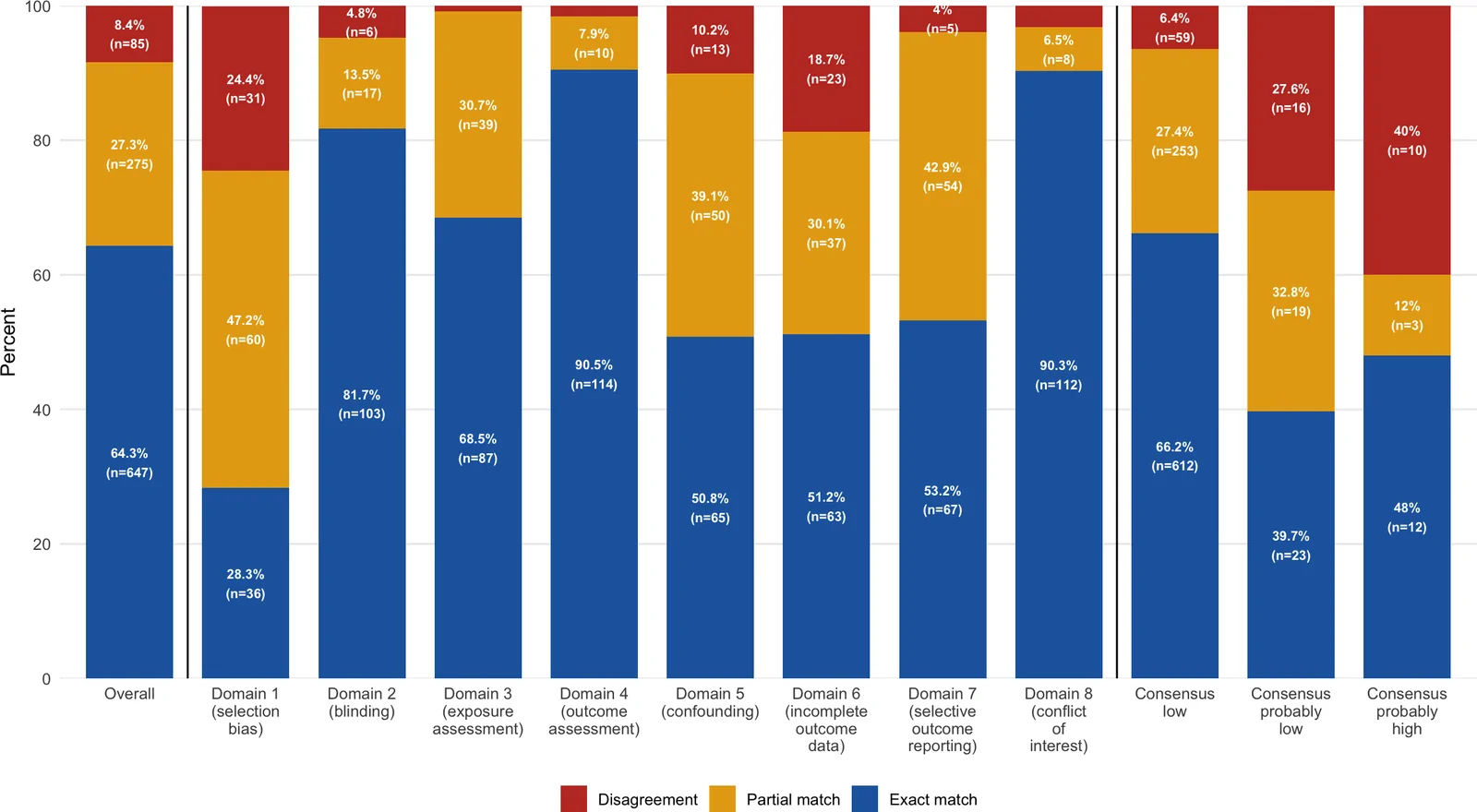
Performance by domain and human risk of bias (RoB) assessment: GPT-5-mini. This figure presents a stacked bar graph of the performance of GPT-5-mini vs human consensus. Blue indicates where the large language model (LLM) matched the consensus exactly, yellow indicates where it matched in direction but not in magnitude, and red indicates where it disagreed in direction. The left bar shows overall performance, the next 8 bars show performance for each domain, and the rightmost 3 bars show performance by human consensus. Note that there were no citations with a human consensus rating of high.

### Performance by Domain

Performance varied substantially across the 8 NavGuide domains (Figure 1). For GPT-5-mini, exact match concordance ranged from 28.1% (36/128) in domain 1 (selection bias) to 90.5% (114/126) in domain 4 (outcome assessment; Figure 1). When considering partial matches, performance was substantially higher across all domains, ranging from 75.6% (domain 1) to 99.2% (domain 3), with several domains showing concordance above 95%. Gemini-3 demonstrated a similar pattern of variability across domains (Multimedia Appendix 1, Figure S2).

### Performance by Human RoB Assessment

LLM performance also varied depending on the human consensus RoB rating. For GPT-5-mini (Figure 1), exact match concordance was highest when human reviewers assigned a consensus rating of “low” RoB (612/924, 66.2% for exact match and 865/924, 93.6% for partial match). Performance was lower for “probably low” ratings (23/58, 39.7% for exact match and 42/58, 72.4% for partial match) and “probably high” ratings (12/25, 48% exact match and 15/25, 60% partial match). Again, Gemini-3 had similar results (Multimedia Appendix 1, Figure S2).

### Consistency Assessment

To assess reproducibility, we ran each paper-domain pair through each LLM 5 times, independently, to ensure that the LLM did not learn from previous runs. The analyses above are from the first run only. Overall, we found moderate consistency across GPT-5-mini runs (Table 4): 62% (632/1019) of assessments yielded identical ratings across all 5 runs (exact match), while 90% (917/1019) showed a consistent direction of assessment (partial match). The highest consistency was in domain 8 (113/126, 90% for exact and 118/126, 94% for partial match) and domain 2 (110/127, 87% for exact and 124/127, 98% for partial). In contrast, domains 1, 6, and 7 showed lower consistency, with exact matches ranging from 32% (41/127) to 47% (60/127). A small percentage of assessments showed substantial variability, with three or more distinct labels across the 5 (4%) runs. At least one run exactly matched the human consensus in 78% of citations; for a partial match, this was true for 94% of citations. We also assessed a majority vote strategy where the label with the most votes was chosen, but this had little impact on performance. Gemini-3 had similar results, both overall and by domain.

**Table 4.** Reproducibility results for each large language model (LLM).^a^

Domain	GPT-5-mini all agree—exact match, n/N (%)	Gemini-3 all agree—exact match, n/N (%)	GPT-5-mini all agree—partial match, n/N (%)	Gemini-3 all agree—partial match, n/N (%)
Overall	632/1019 (62)	602/1021 (59)	917/1019 (90)	971/1021 (95)
1 (selection bias)	41/127 (32)	53/127 (42)	90/127 (71)	109/127 (86)
2 (blinding)	110/127 (87)	55/128 (43)	124/127 (98)	128/128 (100)
3 (exposure assessment)	82/128 (64)	74/128 (58)	124/128 (97)	125/128 (98)
4 (outcome assessment)	106/128 (83)	102/128 (80)	109/128 (85)	128/128 (100)
5 (confounding)	67/128 (52)	61/128 (48)	116/128 (91)	119/128 (93)
6 (incomplete outcome data)	60/127 (47)	72/127 (57)	100/127 (79)	112/127 (88)
7 (selective outcome reporting)	56/128 (44)	67/128 (52)	120/128 (94)	128/128 (100)
8 (conflict of interest)	113/126 (90)	122/127 (96)	118/126 (94)	124/127 (98)
Any LLM label=human consensus	795/1019 (78)	767/1021 (75)	958/1019 (94)	991/1021 (97)
Majority vote=human consensus	652/1019 (64)	552/1021 (54)	907/1019 (89)	981/1021 (96)

a Partial match means agreement in direction but not necessarily in magnitude (eg, low and probably low).

### Secondary Analysis: Full-Text Assessment

We ran a secondary analysis in which we had the LLM identify passages relevant to each RoB domain in the full-text PDF, and then assess the RoB based on the passages it identified. To assess whether the LLM located the same evidence as the human reviewers, we computed the semantic similarity between LLM- and human-selected passages. Passages were embedded using the all-MiniLM-L6-v2 Sentence Transformers model, and similarity was defined as the cosine similarity between embeddings (range −1 to 1; higher values indicate greater similarity in meaning). For each study and RoB domain, every LLM-selected passage was compared with every human-selected passage, and the highest pairwise similarity was retained as the best-match score for that assessment. Assessments in which the humans or the LLM selected no passage were excluded (n=341). The median best-match similarity across assessments was 0.92 for GPT-5-mini (domain-level medians ranged from 0.87 to 0.97). Gemini-3 had similar results. Because this measure reflects only the closest pair of passages for each assessment and captures similarity in meaning rather than verbatim overlap, it indicates whether the LLM identified at least one passage resembling the human-selected evidence. It does not show whether the LLM captured all of the evidence the humans selected, or whether its other selected passages were relevant. Full results for both models are provided in Table S1 of Multimedia Appendix 1. When the LLMs assessed the RoB based on the passages they selected, the results were similar, with GPT-5-mini having a concordance of 74.4% (748/1006) for exact matches (κ=0.204, Gwet AC2=0.917, *F*_1_-score=0.497) and 92.4% (930/1006) for partial matches (κ=0.083, Gwet AC2=0.918, *F*_1_-score=0.961). Similar to the primary analysis, because a large majority of the consensus ratings were low risk, the κ results are artificially reduced by high chance agreement. Gemini-3 performed slightly less well. Full results for both models are provided in Table S2 of Multimedia Appendix 1.

### Qualitative Assessment of GPT-5-Mini Justifications

To identify patterns in why LLMs and humans disagreed, we qualitatively evaluated the “reasoning” provided by both human reviewers and GPT-5-mini. The patterns of discrepancy varied substantially across domains, reflecting the different types of methodological considerations evaluated in each. A subject expert (JL) re-adjudicated all instances where the human and AI ratings disagreed in their direction. This accounted for 8.4% (85/1007) of the total assessed records, ranging from 1% (1/127) for domain 3 to 24% (31/127) for domain 1. After taking both the AI and human justifications into account, the expert assessed whether the rating from the human, the LLM, or neither was correct. Across domains, this assessment agreed with human ratings 47% (40/85) of the time, with those of GPT-5-mini 31% (26/85) of the time, and between the two 22% (19/85) of the time. Table 5 shows the breakdown by domain. This indicates that the LLM ratings caused a change in overall rating in about 4% (43/1007) of studies, with nearly half agreeing with the LLM in direction.

**Table 5.** Post-AI adjudication agreement.^a^

Domain	Number of disagreements, n/N (%)	Post-AI adjudication agreement			Agreement in direction	
		Human, n/N (%)	GPT-5-mini, n/N (%)	Between, n/N (%)	Human, n/N (%)	GPT-5-mini, n/N (%)
Overall	85/1007 (8.4)	40/85 (47.0)	26/85 (30.6)	19/85 (22.4)	47/85 (55.3)	38/85 (44.7)
1 (selection bias)	31/127 (24.4)	11/31 (35.5)	10/31 (32.3)	10/31 (32.3)	15/31 (48.4)	16/31 (51.6)
2 (blinding)	6/126 (4.8)	6/6 (100)	0/6 (0)	0/6 (0)	6/6 (100)	0/6 (0)
3 (exposure assessment)	1/127 (0.8)	0/1 (0)	1/1 (100)	0/1 (0)	0/1 (0)	1/1 (100)
4 (outcome assessment)	2/126 (1.6)	2/2 (100)	0/2 (0)	0/2 (0)	2/2 (100)	0/2 (0)
5 (confounding)	14/128 (10.9)	4/14 (28.6)	5/14 (35.7)	5/14 (35.7)	5/14 (35.7)	9/14 (64.3)
6 (incomplete outcome data)	22/123 (17.9)	13/22 (59.1)	6/22 (27.3)	3/22 (13.6)	14/22 (63.6)	8/22 (36.4)
7 (selective outcome reporting)	5/126 (4.0)	4/5 (80)	1/5 (20)	0/5 (0)	4/5 (80)	1/5 (20)
8 (conflict of interest)	4/126 (3.2)	0/4 (0)	3/4 (75)	1/4 (25)	1/4 (25)	3/4 (75)

a Agreement in direction but not in magnitude, such as low and probably low.

Additionally, approximately 27% (272/1007) of cases were classified as acceptable agreement, ranging from 8% (10/127) in domain 4 to 48% (61/127) in domain 1. Domain 1 (selection bias) had the largest number of discrepant citations, with 61 citations tagged as acceptable agreement. The most common reasons that GPT-5-mini downgraded and the human did not were “potentially unmatched groups,” primarily as a result of the study population being a subset of a larger dataset, and “attrition.” In domain 2 (blinding), there were fewer discrepancies, but almost all were because the LLM downgraded outcomes that were unlikely to be affected by blinding while human reviewers did not. Domain 3 (exposure assessment) and domain 4 (outcome assessment) were dominated by a single tag (quality assurance and quality control not reported), despite the fact that disagreement was much lower in domain 4 (10 citations tagged as acceptable agreement) compared to domain 3 (39 citations tagged as acceptable agreement). In domain 7 (selective outcome reporting), GPT-5-mini downgraded 52 of the 54 citations tagged as acceptable agreement due to the lack of an explicit prespecified protocol. The other domains were not dominated by a single tag, but across domains, it was occasionally unclear to reviewers how the LLM had translated the justification into the RoB it assessed. The full set of tags by domain is presented in Table 3 of Multimedia Appendix 1.

When looking at disagreements, we found the following themes: first, LLMs tended to be stricter than the human rater. The human seemed to assume acceptable methodology even when details were not explicitly reported, whereas the LLM’s justification suggested that it treated this as a potential RoB. Second, based on the justifications, the LLM tended to interpret missing information as resulting in potential bias, while the humans treated it more neutrally. Across domains, an average of 40% of conflicts were due to LLMs downgrading RoB for missing information.

## Discussion

### Principal Findings

Our results suggest that, while the LLMs did not reach the accuracy of human reviewers, they may be useful as a second or third reviewer, specifically highlighting areas in which human reviewers are making potentially inappropriate assessments. The inconsistency across domains and weaker performance for higher-risk studies limit the LLM’s unsupervised usefulness in SR workflows. However, because LLMs were stricter than humans, their assessments may play a role in flagging cases where humans may have been insufficiently strict. Other recent studies, including one by Saadi et al [30], in which 3 LLMs (OpenAI, GPT-5.2 Instant; Google DeepMind, Gemini-3 Fast; and Anthropic, Claude Opus-4‐6) assessed RoB using the ROBINS-I tool, have reported similar findings, concluding that performance is not sufficient for use without human oversight and often differs across domains [31].

Our qualitative assessment suggested that LLMs often missed nuance and context that humans, particularly domain experts, take for granted, often judging actual studies against an ideal study design, which is often impractical. The LLM struggled with missing information in the provided text, which is more common in studies with high RoB. Older studies, including those that evaluated a pretrained machine learning tool for RCT evaluation, RobotReviewer [32], emphasize the importance of linking RoB assessment to specific passages in the text. In this project, we explicitly evaluated whether an LLM could assess RoB when given the correct passages, which is a critical first step to the LLM being able to identify the passages and then assess RoB. The moderate performance on this specific task suggests that LLMs may not yet be ready to perform the broader task of completing the full RoB assessment independently, which is corroborated by the lack of significant improvement when the LLM was given the entire PDF. This finding is corroborated by other studies that have shown that even when given the entire PDF, LLMs had similar accuracy to that reported in this manuscript [17,18].

It is possible that a more specific LLM-prompting strategy would improve consistency across LLM runs. However, developing very detailed prompts takes a great deal of time and effort, and prompts often do not translate across review topics. A generic prompt that is designed to work with existing documentation facilitates more widespread implementation. It is also possible that chaining different LLMs, or even multiple runs of the same LLM, together with some form of adjudication or voting strategy, could improve performance. In our analysis, using a majority vote strategy with the same LLM did not improve performance, but studies have reported that combining LLMs improves performance in other SR tasks, such as in the automation of abstract screening [33].

### Limitations

This study has several limitations. First, we only evaluated a single SR. We are thus unable to generalize our findings to other topics or study designs. However, the premise of the experiment, specifically the use of a general prompt and existing RoB framework, should allow for replication across SR topics and types. Second, the restriction to open-access publications introduces potential selection bias, insofar as open-access publications may differ systematically from non–open-access ones in terms of funding sources, study design, or methodological quality, all of which are factors relevant to RoB. Third, for this review, the preponderance of study-domain pairs was labeled as “low” RoB, with few “probably low” or “probably high” assessments and no “high” assessments. It is possible that the LLMs would perform differently for high RoB studies, but, given the passage-based design (in contrast to including the entire PDF), we do not think this would have substantially changed the results. Fourth, there is some risk of contamination of the model when it is used to evaluate the same data. The Gemini-3 model produces a recitation error when it identifies repeating text from training data, and none were identified, so the risk of contamination is low for that model’s results. However, for GPT-5-mini, there is no indication as to whether it is repeating from training data. Nevertheless, the similar results across models suggest that this may not be a major concern. Fifth, we did not run a full analysis of the Gemini-3 output, and this paper is not designed as a comparison of the two models. We primarily sought to use the Gemini-3 results as a sensitivity analysis for the GPT-5-mini results, but analyzing its justifications might have strengthened our qualitative analysis. Sixth, the qualitative analysis was not blinded, nor did it follow an explicit framework, such as that proposed by Lau et al [34] to assist users in critically assessing AI outputs. Our project predated the publication of this framework, but it may be useful for future research to standardize justification analyses. Moreover, it is important to note that LLM justifications are not validated indicators. Therefore, these results should be interpreted as possible explanations for the discrepant assessments rather than definitive causes. Finally, we did a full analysis of only the studies in which the LLM and the human disagreed in direction (“high” or “probably high” vs “low” or “probably low”). Because we did not re-evaluate the partial matches, we were unable to rerun the analyses with the new gold standard citations. Thus, these results address primarily the utility of incorporating an LLM into the human workflow.

### Conclusions

Although performance was moderate and inconsistent across domains and LLM runs, the results of this study suggest that LLM-based RoB assessment based on human-identified passages may be sufficiently accurate for use with human oversight. LLMs can flag cases for human adjudication or consistency checking, but they are not ready for use as an independent second reviewer. Moreover, the thematic analysis suggests that incorporating LLM adjudications and justifications may improve human RoB assessment through improved consistency.

## Supplementary material

10.2196/99716 Multimedia Appendix 1 Full risk of bias instructions, system prompts, confusion matrices, sensitivity analyses, and detailed results.

## Abbreviations

LLM: large language model
NAS: National Academies of Sciences
PFAS: pre- and polyfluoroalkyl substances
RCT: randomized controlled trial
RoB: risk of bias
ROBINS-E: Risk of Bias in Nonrandomized Studies of Exposure
ROBINS-I: Risk of Bias in Nonrandomized Studies of Interventions
SR: systematic review
