# Supplementary material for: Large Language Model Risk of Bias Assessment for Studies in Environmental Health: Proof-of-Concept Study
Source: JMIR Bioinform Biotechnol. 2026 Nov 9;7:e99716. doi: 10.2196/99716 (PMC13747313; doi:10.2196/99716)
Supplement: Supplementary file 1 [file bioinform-v7-e99716-s001.pdf]

Risk of Bias instructions

This protocol outlines the approach for assessing risk of bias in human observational studies evaluating the relationship between per- and polyfluoroalkyl substances (PFAS) exposure and health outcomes.

<u>Instructions:</u>

- *Please evaluate each individual study for the following nine risk of bias domains. Please assign one of the following ratings for each domain: "low risk," "probably low risk," "probably high risk," "high risk," or "not applicable" and provide details/justification for your rating. If there is empirical evidence or other knowledge that informs the direction of bias, please include this in your answer as well; however, if there is not enough information to do so please do not guess at the direction of bias.*
- *Additionally, please note that some internal validity issues could potentially be appropriately captured in several different risk of bias considerations. In this situation, please select the single most appropriate domain to evaluate this potential bias, to avoid double-counting the same internal validity concern.*

1. Are the study groups at risk of not representing their source populations in a manner that might introduce selection bias?

Assess whether the study population represents the source population. The source population is viewed as the population for which study investigators are targeting their study question of interest. Examples of considerations for this risk of bias domain include: 1) level of detail reported for participant inclusion/exclusion (including details from previously published papers referenced in the article for an existing cohort); 2) participation rates and whether this differed by exposure or outcome group; 3) attrition rates and reasons; and 4) comparisons of study characteristics between the study population and full cohort.

Criteria for a judgment of LOW risk of bias (i.e., answer: "No"):

EITHER:

a) The descriptions of the source population, inclusion/exclusion criteria, recruitment and enrollment procedures, participation and follow-up rates were sufficiently detailed, and adequate data were supplied on the distribution of relevant study sample and population characteristics to support the assertion that risk of selection effects was minimal.

OR

b) Although the descriptions and/or data as indicated in "a" above suggested the potential for selection effects, adequate support was given indicating that potential selection effects were *not* differential across both exposure and outcome.

OR

c) Although the descriptions and/or data as indicated in "a" above suggested the potential for selection effects and there was no support indicating that potential selection effects were *not* differential across both exposure and outcome, selection factors appeared to be well-understood, were measured in the data set, and appropriate adjustment post hoc techniques were used to control for selection bias.

Criteria for the judgment of PROBABLY LOW risk of bias (i.e., answer: "Probably No"):

There is insufficient information about participant selection to permit a judgment of low risk of bias, but there is indirect evidence which suggests that inclusion/exclusion criteria, recruitment and enrollment procedures, and participation and follow-up rates were consistent across groups as described by the criteria for a judgment of low risk of bias.

Criteria for the judgment of PROBABLY HIGH risk of bias (i.e., answer: "Probably Yes"):

There is insufficient information about participant selection to permit a judgment of high risk of bias, but there is indirect evidence which suggests that inclusion/exclusion criteria, recruitment and enrollment procedures, and participation and follow-up rates were inconsistent across groups, as described by the criteria for a judgment of high risk of bias.

Criteria for the judgment of HIGH risk of bias (i.e., answer: "Yes"):

a) There were indications from descriptions of the source population, inclusion/exclusion criteria, recruitment and enrollment procedures, participation and follow-up rates, or data on the distribution of relevant study sample and population characteristics that risk of selection effects were substantial; and
b) There was no support to indicate that potential selection effects were *not* differential across both exposure and outcome; and
c) Adjustment post hoc techniques were not used to control for selection bias.

Criteria for the judgment of NOT APPLICABLE (risk of bias domain is not applicable to study):

There is evidence that participant selection is not an element of study design capable of introducing risk of bias in the study.

2. Was knowledge of the group assignments inadequately prevented (i.e., blinded or masked) during the study, potentially leading to subjective measurement of either exposure or outcome?

Evaluate whether knowledge of the exposure or outcome status was adequately masked among participants, investigators, and outcome assessors. Consider whether blinding would plausibly influence PFAS exposure or outcome measurement.

Criteria for a judgment of LOW risk of bias (i.e., answer: "No"):

Any of the following:

- No blinding, but the review authors judge that the outcome measures as well as the exposure measures are not likely to be influenced by lack of blinding (such as differential

outcome assessment where the outcome is assessed using different measurement or estimation metrics across the exposure groups, or differential exposure assessment where exposure is assessed using different measurement or estimation metrics across the diagnostic or outcome groups); or

- Blinding of key study personnel was ensured, and it is unlikely that the blinding could have been broken; or
- Some key study personnel were not blinded, but exposure and outcome assessment was blinded and the non-blinding of others is unlikely to introduce bias.

Criteria for the judgment of PROBABLY LOW risk of bias (i.e., answer: "Probably No"):

There is insufficient information about blinding to permit a judgment of low risk of bias, but there is indirect evidence which suggests the study was adequately blinded, as described by the criteria for a judgment of low risk of bias. For example, investigators were effectively blinded to the exposure and/or outcome groups if the exposure was measured by a separate entity and the outcome was obtained from a hospital record.

Criteria for the judgment of PROBABLY HIGH risk of bias (i.e., answer: "Probably Yes"):

There is insufficient information about blinding to permit a judgment of high risk of bias, but there is indirect evidence which suggests the study was not adequately blinded, as described by the criteria for a judgment of high risk of bias.

Criteria for the judgment of HIGH risk of bias (i.e., answer: "Yes"):

Any of the following:

- No blinding or incomplete blinding, and the outcome measures or exposure measures is likely to be influenced by lack of blinding (i.e., differential outcome or exposure assessment); or
- Blinding of key study personnel attempted, but likely that the blinding could have been broken so as to introduce bias; or
- Some key study personnel were not blinded, and the non-blinding of others was likely to introduce bias.

Criteria for the judgment of NOT APPLICABLE (risk of bias domain is not applicable to study):

There is evidence that blinding is not an element of study design capable of introducing risk of bias in the study.

3. Were exposure assessment methods lacking accuracy?

Assess the validity and reliability of PFAS exposure measures, including:

- Biomonitoring data (e.g., serum PFAS levels)
- Environmental models (e.g., residential proximity to PFAS-contaminated sites)
- Surrogates (e.g., occupational/job titles or self-report).

Consider the temporal accuracy, spatial resolution, and exposure window relevance.

Criteria for a judgment of LOW risk of bias (i.e., answer: "No"):

The reviewers judge that there is low risk of exposure misclassification, i.e.:

- There is high confidence in the accuracy of the exposure assessment methods, such as methods that have been tested for validity and reliability in measuring the targeted exposure; or
- Less-established or less direct exposure measurements are validated against well-established or direct methods;

AND if applicable (e.g. for laboratory measurements), appropriate QA/QC for methods are described and are satisfactory, with at least three of the following items reported, or at least two of the following items reported plus evidence of satisfactory performance in a high quality inter-laboratory comparison:

- Limit of detection or quantification;
- standards recovery;
- measure of repeatability;
- investigation and prevention of blanks contamination.

Criteria for the judgment of PROBABLY LOW risk of bias (i.e., answer: "Probably No"):

There is insufficient information about the exposure assessment methods to permit a judgment of low risk of bias, but there is indirect evidence that suggests that methods were robust, as described by the criteria for a judgment of low risk of bias. Studies only reporting that the QA/QC items above were satisfactory but not reporting all of the actual numbers may receive a judgment of "probably low risk of bias."

Criteria for the judgment of PROBABLY HIGH risk of bias (i.e., answer: "Probably Yes"):

There is insufficient information about the exposure assessment methods to permit a judgment of high risk of bias, but there is indirect evidence that suggests that methods were not robust, as described by the criteria for a judgment of high risk of bias

Criteria for the judgment of HIGH risk of bias (i.e., answer: "Yes"):

The reviewers judge that there is high risk of exposure misclassification and any one of the following:

- There is low confidence in the accuracy of the exposure assessment methods; or
- Less-established or less direct exposure measurements are not validated and are suspected to introduce bias that impacts the outcome assessment (example: participants are asked to report exposure status retrospectively, subject to recall bias); or
- Uncertain how exposure information was obtained

Criteria for the judgment of NOT APPLICABLE (risk of bias domain is not applicable to study):

There is evidence that exposure assessment methods are not capable of introducing risk of bias in the study.

4. Were outcome assessment methods lacking accuracy?

Evaluate whether health outcomes were measured accurately, consistently, and using validated methods. Consider differential outcome assessment across exposure groups.

Criteria for a judgment of LOW risk of bias (i.e., answer: "No"):

The reviewers judge that there is low risk of outcome misclassification, i.e.:

- Outcomes were assessed and defined consistently across all study participants, using valid and reliable measures; or
- Less-established or less direct outcome measurements are validated against well-established or direct methods; or
- Appropriate sensitivity analyses were conducted that suggest the influence of outcome misclassification would be minimal
- AND, if applicable, appropriate QA/QC for methods is described and is satisfactory.

Criteria for the judgment of PROBABLY LOW risk of bias (i.e., answer: "Probably No"):

There is insufficient information about the outcome assessment methods to permit a judgment of low risk of bias, but there is indirect evidence which suggests that methods were robust, as described by the criteria for a judgment of low risk of bias. Appropriate QA/QC for methods are not described but the review authors judge that the outcome and the outcome assessment are objective and uniform across study groups.

Criteria for the judgment of PROBABLY HIGH risk of bias (i.e., answer: "Probably Yes"):

There is insufficient information about the outcome assessment methods to permit a judgment of high risk of bias, but there is indirect evidence which suggests that methods were not robust, as described by the criteria for a judgment of high risk of bias.

Criteria for the judgment of HIGH risk of bias (i.e., answer: "Yes"):

The reviewers judge that there is high risk of outcome misclassification and any one of the following:

- There is low confidence in the accuracy of the outcome assessment methods; or
- Less-established or less direct outcome measurements are not validated and are suspected to introduce bias that impacts the outcome assessment
- Uncertain how outcome information was obtained

Criteria for the judgment of NOT APPLICABLE (risk of bias domain is not applicable to study):

There is evidence that outcome assessment methods are not capable of introducing risk of bias in the study.

5. Was potential confounding inadequately incorporated?

Assess whether studies accounted for key confounders relevant to PFAS exposure and the specific outcome. Tier I confounders may include age, sex, smoking, socioeconomic status, and occupation. Tier II confounders may include diet, race/ethnicity, BMI, and comorbidities.

Criteria for a judgment of LOW risk of bias (i.e., answer: “No”):

> The study appropriately assessed and accounted for (i.e., matched, stratified, excluded certain populations (i.e., smokers) or statistically controlled for) all important confounders (Tier I) using appropriate statistical techniques, or reported that important confounders were evaluated and omitted because inclusion did not substantially affect the results. The determination of specific confounders may also be informed by, but not limited to, the studies included in the overall review,
>
> AND the study appropriately assessed and accounted for (i.e., matched, stratified, or statistically controlled for) other potentially important confounders relevant (Tier II) using appropriate statistical techniques, or reported that these confounders were evaluated and omitted because inclusion did not substantially affect the results,
>
> AND the important potential confounders were measured consistently across study groups using valid and reliable methods, or the influence of covariate measurement error was determined, through sensitivity analysis, to be minimal.

Criteria for the judgment of PROBABLY LOW risk of bias (i.e., answer: “Probably No”):

> The study appropriately accounted for most but not all of the important confounders (Tier I) or used appropriate statistical techniques;
>
> AND some of the other potentially important confounders relevant (Tier II) using appropriate statistical techniques,
>
> OR reported that these confounders were evaluated and omitted because inclusion did not substantially affect the results;
>
> AND this is not expected to introduce substantial bias.

Criteria for the judgment of PROBABLY HIGH risk of bias (i.e., answer: “Probably Yes”):

> The study evaluated some but not all of the important confounders (Tier I),
>
> AND some but not all of the other potentially important confounders relevant (Tier II),
>
> OR used questionable statistical techniques for confounder adjustment;
>
> AND this is expected to introduce substantial bias.

Criteria for the judgment of HIGH risk of bias (i.e., answer: “Yes”):

> The study did not account for or evaluate multiple important confounders (Tier I),
>
> AND did not account for or evaluate multiple other potentially important confounders relevant (Tier II),

OR the important potential confounders were inappropriately measured and/or inappropriately analyzed across study groups.

Criteria for the judgment of NOT APPLICABLE (risk of bias domain is not applicable to study):

There is evidence that outcome assessment methods are not capable of introducing risk of bias in the study.

6. Were incomplete outcome data inadequately addressed?

Assess the extent and handling of missing outcome data. Consider loss to follow-up, differences in attrition by exposure group, and statistical imputation methods used.

Criteria for a judgment of LOW risk of bias (i.e., answer: "No"):

Participants were followed long enough to obtain outcome measurements
OR any one of the following:

- No missing outcome data; or
- Reasons for missing outcome data unlikely to be related to true outcome (for survival data, censoring unlikely to introduce bias); or
- Attrition or missing outcome data balanced in numbers across exposure groups, with similar reasons for missing data across groups; or
- For dichotomous outcome data, the proportion of missing outcomes compared with observed event risk not enough to have a relevant impact on the exposure effect estimate; or
- For continuous outcome data, plausible effect size (difference in means or standardized difference in means) among missing outcomes not enough to have a relevant impact on the observed effect size; or
- Missing data have been imputed using appropriate methods

Criteria for the judgment of PROBABLY LOW risk of bias (i.e., answer: "Probably No"):

There is insufficient information about incomplete outcome data to permit a judgment of low risk of bias, but there is indirect evidence which suggests incomplete outcome data was adequately addressed, as described by the criteria for a judgment of low risk of bias.

Criteria for the judgment of PROBABLY HIGH risk of bias (i.e., answer: "Probably Yes"):

There is insufficient information about incomplete outcome data to permit a judgment of high risk of bias, but there is indirect evidence which suggests incomplete outcome data was not adequately addressed, as described by the criteria for a judgment of high risk of bias.

Criteria for the judgment of HIGH risk of bias (i.e., answer: "Yes"):

Participants were not followed long enough to obtain outcome measurements
OR any one of the following:

- Reason for missing outcome data likely to be related to true outcome, with either imbalance in numbers or reasons for missing data across exposure groups; or
- For dichotomous outcome data, the proportion of missing outcomes compared with observed event risk enough to induce biologically relevant bias in intervention effect estimate; or
- For continuous outcome data, plausible effect size (difference in means or standardized difference in means) among missing outcomes enough to induce biologically relevant bias in observed effect size; or
- Potentially inappropriate application of imputation.

Criteria for the judgment of NOT APPLICABLE (risk of bias domain is not applicable to study):

> There is evidence that incomplete outcome data is not capable of introducing risk of bias in the study.

7. Does the study report appear to have selective outcome reporting?

Evaluate whether all prespecified outcomes were reported consistently with study protocols or stated objectives. Look for evidence of outcome switching or incomplete reporting.

Criteria for a judgment of LOW risk of bias (i.e., answer: “No”):

> All of the study’s pre-specified (primary and secondary) outcomes outlined in the pre-published protocol or the published manuscript’s methods, abstract, and/or introduction section that are of interest in the review have been reported in the pre-specified way.

Criteria for the judgment of PROBABLY LOW risk of bias (i.e., answer: “Probably No”):

> There is insufficient information about selective outcome reporting to permit a judgment of low risk of bias, but there is indirect evidence which suggests the study was free of selective reporting, as described by the criteria for a judgment of low risk of bias. This includes if a pre-published protocol is not available but the study’s pre-specified (primary and secondary) outcomes outlined in the published manuscript’s methods, abstract, and/or introduction section that are of interest in the review have been reported in the pre-specified way.

Criteria for the judgment of PROBABLY HIGH risk of bias (i.e., answer: “Probably Yes”):

> There is insufficient information about selective outcome reporting to permit a judgment of high risk of bias, but there is indirect evidence which suggests the study was not free of selective reporting, as described by the criteria for a judgment of high risk of bias. This includes if a pre-published protocol is not available and the study’s pre-specified (primary and secondary) outcomes outlined in the published manuscript’s methods, abstract, and/or introduction section that are of interest in the review have not been reported in the pre-specified way.

Criteria for the judgment of HIGH risk of bias (i.e., answer: “Yes”):

> Any one of the following:

- Not all of the study's pre-specified primary outcomes (as outlined in the pre-published protocol or published manuscript's methods, abstract, and/or introduction) have been reported; or
- One or more primary outcomes is reported using measurements, analysis methods or subsets of the data (e.g. subscales) that were not pre-specified; or
- One or more reported primary outcomes were not pre-specified (unless clear justification for their reporting is provided, such as an unexpected effect); or
- One or more outcomes of interest are reported incompletely

Criteria for the judgment of NOT APPLICABLE (risk of bias domain is not applicable to study):

> There is evidence that selective outcome reporting is not capable of introducing risk of bias in the study.

8. Did the study receive any support from a company, study author, or other entity having a financial interest in any of the exposures studied?

Determine whether the study was funded by or involved entities with financial or vested interests in PFAS exposure outcomes. Evaluate author affiliations and funding disclosures.

Criteria for a judgment of LOW risk of bias (i.e., answer: "No"):

> The study did not receive support from a company, study author, or other entity having a financial interest in the outcome of the study. Examples include the following:
> - Funding source is limited to government, non-profit organizations, or academic grants funded by government, foundations and/or non-profit organizations;
> - Chemicals or other treatment used in study were purchased from a supplier;
> - Company affiliated staff are not mentioned in the acknowledgements section;
> - Authors were not employees of a company with a financial interest in the outcome of the study;
> - Company with a financial interest in the outcome of the study was not involved in the design, conduct, analysis, or reporting of the study and authors had complete access to the data;
> - Study authors make a claim denying conflicts of interest;
> - Study authors are unaffiliated with companies with financial interest, and there is no reason to believe a conflict of interest exists;
> - All study authors are affiliated with a government agency (are prohibited from involvement in projects for which there is a conflict of interest or an appearance of conflict of interest).

Criteria for the judgment of PROBABLY LOW risk of bias (i.e., answer: "Probably No"):

> There is insufficient information to permit a judgment of low risk of bias, but there is indirect evidence which suggests the study was free of support from a company, study author, or other entity having a financial interest in the outcome of the study, as described by the criteria for a judgment of low risk of bias.

Criteria for the judgment of PROBABLY HIGH risk of bias (i.e., answer: "Probably Yes"):

There is insufficient information to permit a judgment of high risk of bias, but there is indirect evidence which suggests the study was not free of support from a company, study author, or other entity having a financial interest in the outcome of the study, as described by the criteria for a judgment of high risk of bias.

Criteria for the judgment of HIGH risk of bias (i.e., answer: "Yes"):

The study received support from a company, study author, or other entity having a financial interest in the outcome of the study. Examples of support include:

- Research funds;
- Chemicals, equipment or testing provided at no cost;
- Writing services;
- Author/staff from study was employee or otherwise affiliated with company with financial interest;
- Company limited author access to the data;
- Company was involved in the design, conduct, analysis, or reporting of the study;
- Study authors claim a conflict of interest

Criteria for the judgment of NOT APPLICABLE (risk of bias domain is not applicable to study):

There is evidence that conflicts of interest are not capable of introducing risk of bias in the study.

9. Did the study appear to have other problems that could put it at a risk of bias?

Capture other potential sources of bias not covered above.

Criteria for a judgment of LOW risk of bias (i.e., answer: "No"):

The study appears to be free of other sources of bias.

Criteria for the judgment of PROBABLY LOW risk of bias (i.e., answer: "Probably No"):

There is insufficient information to permit a judgment of low risk of bias, but there is indirect evidence which suggests the study was free of other threats to validity.

Criteria for the judgment of PROBABLY HIGH risk of bias (i.e., answer: "Probably Yes"):

There is insufficient information to permit a judgment of high risk of bias, but there is indirect evidence which suggests the study was not free of other threats to validity, as described by the criteria for a judgment of high risk of bias.

Criteria for the judgment of HIGH risk of bias (i.e., answer: "Yes"):

There is at least one important risk of bias. For example, the study:

- Had a potential source of bias related to the specific study design used; or
- Stopped early due to some data-dependent process (including a formal-stopping rule); or
- The conduct of the study is affected by interim results (e.g. recruiting additional participants from a subgroup showing greater or lesser effect); or
- Has been claimed to have been fraudulent; or
- Had some other problem

1. System Prompt:
"You are an advanced research assistant tasked with evaluating questions related to the assessment of bias. Your function is to analyze queries about the randomization and blinding processes, participant awareness, and statistical analysis. For each question, your response should follow its instructions. Focus on the overall picture rather than looking for red flags when assessing the risk of bias. Also take into account the Risk of Bias Instructions when making the assessment."

2. User Prompt Format:

```
[
        "ROB Instructions:",
        Images from ROB Instructions PDF,
        "
        {
                "section": Rob domain the query is a part of,
                "question": query ,
                "passages": passages
        }
        ",
]
```

3. Output Format:
Structured Query to ensure it returns answer in a specific format specified in the schema.

```
schema = {
    'properties': {
        'answer': {
            'description': 'Chosen answer between the given choices.',
            'enum': ['Not Applicable', 'Low Risk', 'Probably Low Risk', 'High Risk', 'Probably High Risk'],
            'title': 'Answer',
            'type': 'string'
        },
        'justification': {
            'description': 'Justify why the given answer was chosen.',
            'title': 'Justification',
            'type': 'string'
        }
    },
    'required': ['answer', 'justification'],
    'title': 'Answer',
    'type': 'object'
}
```

4. Model Settings:
4a. Completions API:
OpenAI has two types of API. Completions API the older API format and Responses API the newer API format. Completions API was chosen because we wanted the Gemini OpenAI compatibility layer (a tool to let us run Gemini using the same API as OpenAI) only supports the Completions API. This does not change the results as we did not need to use any of the additional features in the Responses API.

4b. Gpt-5-mini Default Parameters
I could not find any official documentation showing what the default parameters are:
https://community.openai.com/t/whats-the-default-verbosity-and-reasoning-for-gpt-5-model/1357054
https://developers.openai.com/api/docs/models/gpt-5-mini
temperature = 1.0 (required for reasoning models)
top_p = 1.0 (required for reasoning models)
reasoning_effort = “medium”
verbosity = “medium”
max_output_tokens = 128,000

4c. Gemini-3-flash-preview Default Parameters
https://docs.cloud.google.com/gemini-enterprise-agent-platform/models/gemini/3-flash
https://ai.google.dev/gemini-api/docs/gemini-3
temperature = 1.0
thinking_level = “high”
top_p = 0.95
top_k = 64 (not supported in OpenAI only in Gemini)
max_output_tokens = 65,536

5. Miscellaneous:
The code will call the API and if it fails it will retry, this can only happen a maximum of 3 times. There is no malformed responses as the structured query forces the answer to be in one of the multiple choice. There is no handling for the justification given since the max output token is allowed it could potentially use all of it, but that is unlikely to happen. There was no manual correction as structured query ensured answers fit the required structure.

6. API Version:
Gemini API Version: “v1beta”
Azure OpenAI API Version: “2024-12-01-preview”

7. Access Date:

January 2026

8. Model Costs:

8a. Gpt-5-mini cost:

Input = $0.25

Cached = $0.03

Output = $10

8b. Gemini-3-flash-preview cost:

Input = $0.50

Cached = $0.05

Output = $3

Confusion Matrices

GPT (vertical) vs Human Consensus (horizontal): Exact Match

	Low Risk	Probably Low Risk	Probably High Risk	High Risk	Not Applicable
Low Risk	612	19	5	0	0
Probably Low Risk	253	23	5	0	0
Probably High Risk	29	9	12	0	0
High Risk	26	7	3	0	0
Not Applicable	4	0	0	0	0

Gemini (vertical) vs Human Consensus (horizontal): Exact Match

	Low Risk	Probably Low Risk	Probably High Risk	High Risk	Not Applicable
Low Risk	471	14	6	0	0
Probably Low Risk	435	34	10	0	0
Probably High Risk	17	10	6	0	0
High Risk	1	0	3	0	0
Not Applicable	0	0	0	0	0

Human 1 (vertical) vs Human Consensus (horizontal): Exact Match

Predicted	Low Risk	Probably Low Risk	Probably High Risk	High Risk	Not Applicable
Low Risk	840	27	12	0	0
Probably Low Risk	78	31	3	0	0
Probably High Risk	5	0	10	0	0
High Risk	1	0	0	0	0
Not Applicable	0	0	0	0	0

Human 2 (vertical) vs Human Consensus (horizontal): Exact Match

Predicted	Low Risk	Probably Low Risk	Probably High Risk	High Risk	Not Applicable
Low Risk	869	20	8	0	0
Probably Low Risk	53	37	4	0	0
Probably High Risk	1	0	13	0	0
High Risk	0	1	0	0	0

Predicted	Low Risk	Probably Low Risk	Probably High Risk	High Risk	Not Applicable
Not Applicable	1	0	0	0	0

GPT-5 (vertical) vs Human Consensus (horizontal) LLM identify passages

Predicted	Low Risk	Probably Low Risk	Probably High Risk	High Risk	Not Applicable
Low Risk	733	26	11	0	0
Probably Low Risk	151	15	9	0	0
Probably High Risk	16	7	0	0	0
High Risk	23	10	5	0	0
Not Applicable	0	0	0	0	0

Gemini (vertical) vs Human Consensus (horizontal) LLM identify passages

Predicted	Low Risk	Probably Low Risk	Probably High Risk	High Risk	Not Applicable
Low Risk	544	19	6	0	0
Probably Low Risk	253	25	10	0	0
Probably High Risk	12	6	5	0	0
High Risk	2	1	0	0	0
Not Applicable	0	0	0	0	0

GPT (vertical) vs Human Consensus (horizontal): Partial Match

	Low Risk	High Risk
Low Risk	907	10
High Risk	71	15

Gemini (vertical) vs Human Consensus (horizontal): Partial Match

	Low Risk	High Risk
Low Risk	954	16
High Risk	28	9

Human 1 (vertical) vs Human Consensus (horizontal): Partial Match

	Low Risk	High Risk
Low Risk	976	15
High Risk	6	10

Human 2 (vertical) vs Human Consensus (horizontal): Partial Match

	Low Risk	High Risk
Low Risk	979	12
High Risk	2	13

GPT-5 (vertical) vs Human Consensus (horizontal) LLM identify passages: Partial Match

	Low Risk	High Risk
Low Risk	925	20
High Risk	56	5

Gemini (vertical) vs Human Consensus (horizontal) LLM identify passages: Partial Match

	Low Risk	High Risk
Low Risk	841	16
High Risk	21	5

Figure S1: Differential conservativeness

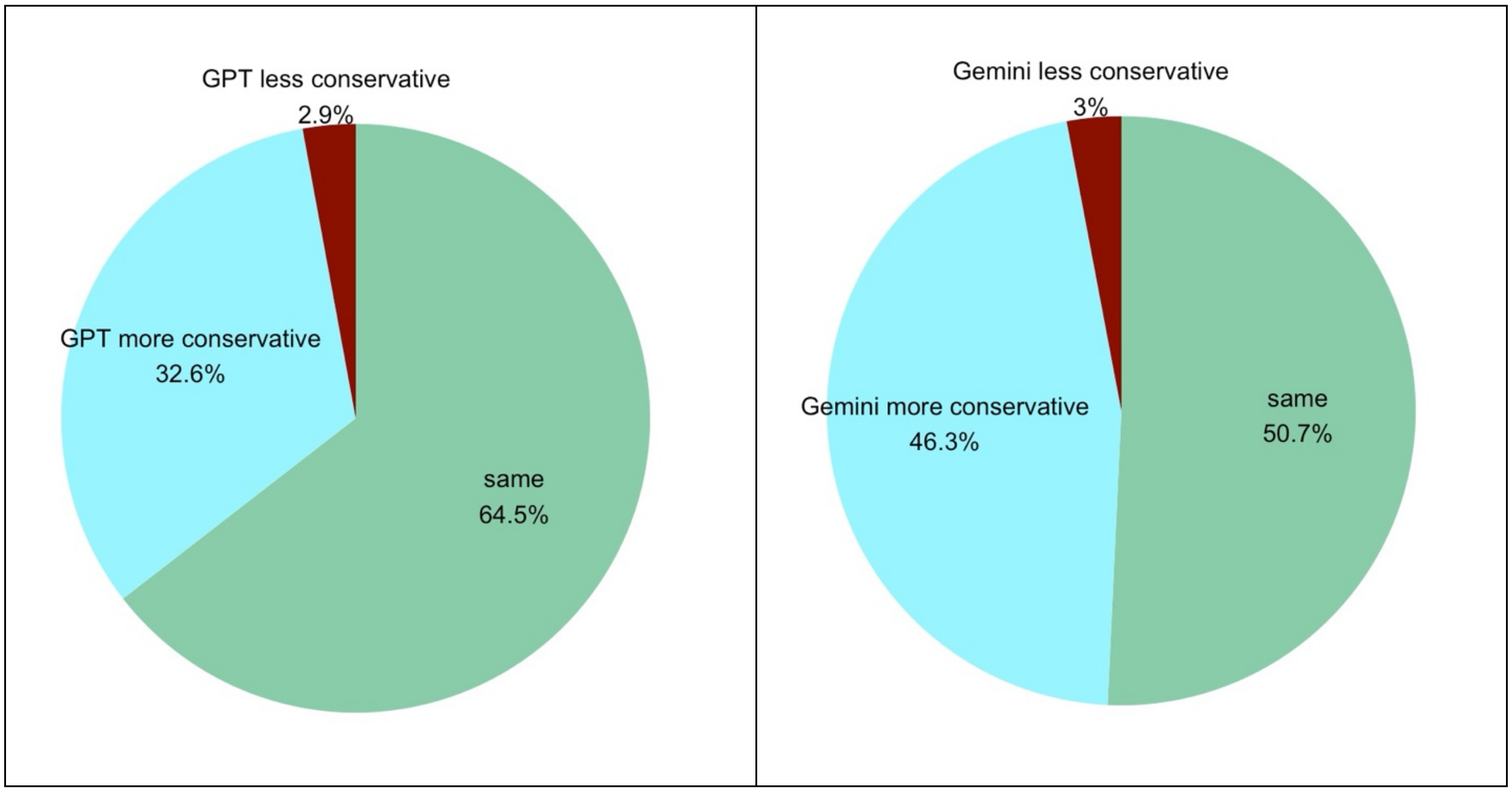

Figure S2: Gemini Percent Concordance

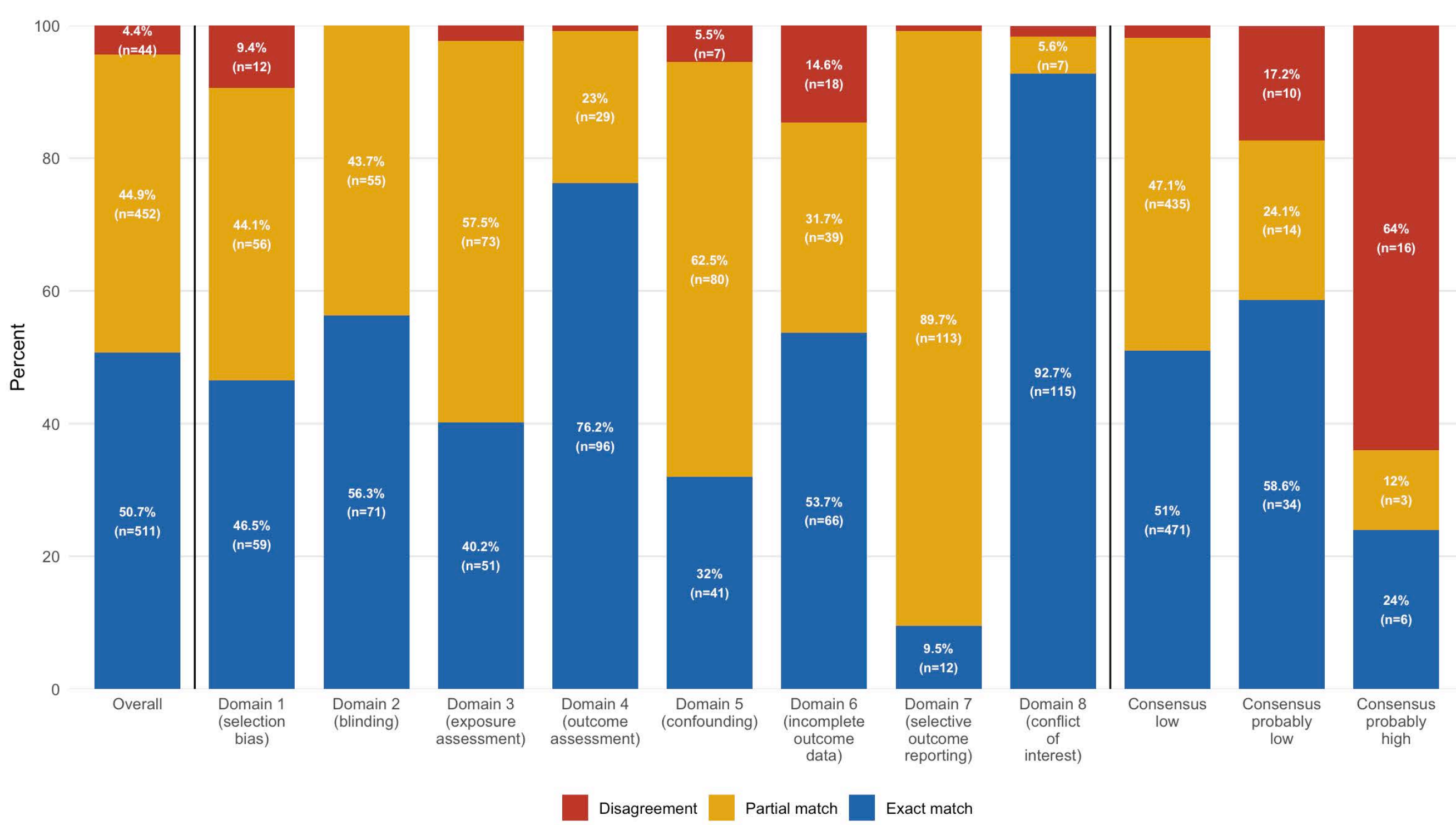

Sensitivity analysis: full-text assessment

Table S1: proportion of text identified by the LLM that matches that identified by the human.

Domain	GPT-5		Gemini	
	Median	IQR	Median	IQR
Overall	0.92	0.81-0.98	0.90	0.71-1.00
1 (selection bias)	0.93	0.88- 0.97	0.84	0.07-0.99
2 (blinding)	0.87	0.78-0.94	0.90	0.70-1.00
3 (exposure assessment)	0.92	0.78-0.98	0.89	0.73-0.99
4 (outcome assessment)	0.93	0.86-0.98	0.88	0.69-0.98
5 (confounding)	0.93	0.86-0.97	0.95	0.77-1.00
6 (incomplete outcome data)	0.84	0.61-0.97	0.86	0.44-1.00
7 (selective outcome reporting)	0.93	0.78-0.99	0.88	0.75-1.00
8 (conflict of interest)	0.97	0.89-0.99	0.99	0.94-1.00

Table S2: Results when passages are identified by the LLM

Comparison	N	Percent concordance (95% CI)	Gwet's AC2 (95% CI)	Cohens kappa (95% CI)	F1 Score (95% CI)
Exact Match					
GPT-5	1,006	74.4<br>(71.7, 77.1)	0.917<br>(0.901,0.933)	0.204<br>(0.131, 0.278)	0.497<br>(0.467, 0.532)
Gemini	883	65.0<br>(61.9, 68.2)	0.927<br>(0.917,0.938)	0.187<br>(0.116, 0.258)	0.388<br>(0.329, 0.449)
Partial Match					
GPT-5	1,006	92.4<br>(90.8, 94.1)	0.918<br>(0.899, 0.937)	0.083<br>(-0.010, 0.178)	0.961<br>(0.951, 0.969)
Gemini	883	95.8<br>(94.5, 97.1)	0.956<br>(0.941, 0.970)	0.191<br>(0.033, 0.350)	0.978<br>(0.971, 0.985)

Table S3: Tags based on review of "Acceptable Agreement" human justifications and thinking trace

Tag	N with Tag	Acceptable Agreement, N total	Percentage
**Domain 1 (selection bias)**			
potentially unmatched groups	20	61	32.8
attrition	19	61	31.1
insufficient information	14	61	23
generalizability	3	61	4.9
sample selection	3	61	4.9
complete cases	2	61	3.3
**Domain 2 (blinding)**			
downgraded despite outcome unlikely to be affected by blinding	7	17	41.2
failure to translate RoB	6	17	35.3
human error	3	17	17.6
insufficient information	1	17	5.9
**Domain 3 (exposure assessment)**			
QA/QC not reported	35	39	89.7
AI missed insufficient information	1	39	2.6
human error	1	39	2.6
insufficient information	1	39	2.6
temporal relevance	1	39	2.6
**Domain 4 (outcome assessment)**			
QA/QC not reported	7	10	70
gestational age measurement	1	10	10
possible human error	1	10	10
possible measurement error	1	10	10
**Domain 5 (confounding)**			
possible residual confounding	22	49	44.9
missing confounders	21	49	42.9
missing data	2	49	4.1
possible incorrect adjustment	2	49	4.1
unclear what was missed	2	49	4.1
insufficient information	23	37	62.2
**Domain 6 (incomplete outcome data)**			
unclear how rationale translates to RoB	4	37	10.8
missing outcome data	3	37	8.1
minimal incomplete outcome data	2	37	5.4
single imputation	2	37	5.4
restriction to complete cases	1	37	2.7

Tag	N with Tag	Acceptable Agreement, N total	Percentage
selection bias	1	37	2.7
some variables have very high missingness	1	37	2.7
**Domain 7 (selective outcome reporting)**			
lack of pre-specified protocol	30	54	55.6
lack of pre-specified protocol or outcome pre-specification	22	54	40.7
selective reporting possible	1	54	1.9
unclear what was missed	1	54	1.9
**Domain 8 (conflict of interest)**			
AI identified COI	3	8	37.5
AI missed COI	3	8	37.5
govt agency misclassified as industry	1	8	12.5
insufficient information	1	8	12.5
